# Effects of Potassium or Sodium Supplementation on Mineral Homeostasis: A Controlled Dietary Intervention Study

**DOI:** 10.1210/clinem/dgaa359

**Published:** 2020-06-07

**Authors:** Jelmer K Humalda, Stanley M H Yeung, Johanna M Geleijnse, Lieke Gijsbers, Ineke J Riphagen, Ewout J Hoorn, Joris I Rotmans, Liffert Vogt, Gerjan Navis, Stephan J L Bakker, Martin H de Borst

**Affiliations:** 1 Department of Internal Medicine, Division of Nephrology, University of Groningen, University Medical Center Groningen, RB Groningen, the Netherlands; 2 Division of Human Nutrition and Health, Wageningen University, HB Wageningen, the Netherlands; 3 Department of Laboratory Medicine, University of Groningen, University Medical Center Groningen, RB Groningen, the Netherlands; 4 Department of Internal Medicine, Division of Nephrology & Transplantation, Erasmus Medical Center, University Medical Center Rotterdam, CA Rotterdam, The Netherlands; 5 Department of Internal Medicine, Leiden University Medical Center, RC Leiden, the Netherlands; 6 Department of Internal Medicine, Section of Nephrology, Amsterdam Cardiovascular Sciences, Amsterdam University Medical Centers, University of Amsterdam, DD Amsterdam Zuidoost, the Netherlands

**Keywords:** Diet controlled clinical trial, nutrition, fibroblast growth factor 23, calcium-phosphate metabolism, potassium, sodium

## Abstract

**Context:**

Although dietary potassium and sodium intake may influence calcium-phosphate metabolism and bone health, the effects on bone mineral parameters, including fibroblast growth factor 23 (FGF23), are unclear.

**Objective:**

Here, we investigated the effects of potassium or sodium supplementation on bone mineral parameters.

**Design, setting, participants:**

We performed a post hoc analysis of a dietary controlled randomized, blinded, placebo-controlled crossover trial. Prehypertensive individuals not using antihypertensive medication (n = 36) received capsules containing potassium chloride (3 g/d), sodium chloride (3 g/d), or placebo. Linear mixed-effect models were used to estimate treatment effects.

**Results:**

Potassium supplementation increased plasma phosphate (from 1.10 ± 0.19 to 1.15 ± 0.19 mmol/L, *P* = 0.004), in line with an increase in tubular maximum of phosphate reabsorption (from 0.93 ± 0.21 to 1.01 ± 0.20 mmol/L, *P *< 0.001). FGF23 decreased (114.3 [96.8-135.0] to 108.5 [93.5-125.9] RU/mL, *P* = 0.01), without change in parathyroid hormone and 25-hydroxy vitamin D_3_. Fractional calcium excretion decreased (from 1.25 ± 0.50 to 1.11 ± 0.46 %, *P* = 0.03) without change in plasma calcium. Sodium supplementation decreased both plasma phosphate (from 1.10 ± 0.19 to 1.06 ± 0.21 mmol/L, *P* = 0.03) and FGF23 (from 114.3 [96.8-135.0] to 108.7 [92.3-128.1] RU/mL, *P* = 0.02). Urinary and fractional calcium excretion increased (from 4.28 ± 1.91 to 5.45 ± 2.51 mmol/24 hours, *P *< 0.001, and from 1.25 ± 0.50 to 1.44 ± 0.54 %, *P* = 0.004, respectively).

**Conclusions:**

Potassium supplementation led to a decrease in FGF23, which was accompanied by increase in plasma phosphate and decreased calcium excretion. Sodium supplementation reduced FGF23, but this was accompanied by decrease in phosphate and increase in fractional calcium excretion. Our results indicate distinct effects of potassium and sodium intake on bone mineral parameters, including FGF23.

**Clinical Trial Registration number:**

NCT01575041

The Western diet is characterized by a high sodium and low potassium content (1), and it has been associated with noncommunicable diseases such as hypertension, cardiovascular, chronic kidney, and mineral and bone disorders (2–4). More specifically, high intake of sodium and low intake of potassium have been linked with an increased risk of cardiovascular disease and mortality (5–8). Mechanistically, these associations are likely at least in part mediated by blood pressure, but additional factors may be involved.

Deregulations in bone and mineral metabolism, including hyperphosphatemia, 25-hydroxy vitamin D_3_ (25[OH]-vitamin D_3_) deficiency, hyperparathyroidism, and high levels of the phosphaturic hormone fibroblast growth factor 23 (FGF23), have been associated with adverse outcomes in various populations (9–14). 25[OH]-vitamin D_3_ is converted to biological active 1,25[OH]2-vitamin D_3_ predominantly in the kidneys by 1-α-hydroxylase. Active vitamin D stimulates calcium and phosphate reabsorption in the gut, thus increasing plasma calcium. 25[OH]-vitamin D_3_ deficiency may lead to decreased plasma calcium, which triggers PTH production (15, 16). PTH increases bone resorption of calcium, suppresses renal phosphate reabsorption, and increases conversion of vitamin D. FGF23 inhibits renal phosphate reabsorption and 1-α-hydroxylase, inhibiting conversion of 25[OH]-vitamin D_3_ to 1,25[OH]2-vitamin D_3_ (17). Vitamin D, PTH, and FGF23 are part of intertwined feedback loops regulating the calcium and phosphate balance (18–20).

FGF23 is more and more identified to be a cardiovascular-related detrimental factor (21–23), and several strategies to reduce FGF23 levels have been studied extensively (24). Interestingly, recent studies suggest that lower potassium intake is associated with a higher FGF23 level (25) and that changes in potassium or sodium homeostasis may influence bone and mineral parameters and bone health (26–29). A study found that a varying amount of salt and a Dietary Approaches to Stop Hypertension diet, which is among other things high in potassium, could improve bone turnover markers and calcium metabolism (30). However, the specific effects of altered potassium or sodium intake on bone and mineral parameters, and particularly calcium/phosphate-regulating hormones including FGF23, in humans remain unclear.

Here, we performed a post hoc analysis in a dietary controlled randomized, blinded, placebo-controlled crossover trial in prehypertensive individuals. In the current study, we investigated the effects of sodium or potassium supplementation, in the context of a controlled diet, on bone and mineral parameters.

## Subjects and Methods

### Study design

We analyzed a double-blinded, randomized, placebo-controlled, crossover study that assessed the effects of both potassium and sodium supplementation on blood pressure and vascular function in untreated prehypertensive individuals (i.e., individuals with a morning office systolic blood pressure [SBP] between 130 and 159 mm Hg after an overnight fasting) who did not use antihypertensive medication. The study protocol has been extensively described before (31). In brief, the participants were provided with a controlled diet, which contained on average 2.4 g (104 mmol) of sodium, based on the recommended maximum sodium intake of 2.0 to 2.4 g per day (which equals 87-104 mmol sodium or 5-6 g salt per day), and 2.3 g (59 mmol) of potassium per day for a 2500-kcal intake. The research facility supplied 90% of the daily energy needs, the remaining 10% were chosen by the participants from a list of products that were low in sodium and potassium. The average composition of the diet was calculated for which nutrient values were obtained from the Dutch food composition table (32), these values were described in a previously published work (31). For this study, we calculated the average phosphorus intake of the diet which was 2004 mg/d and standardized to 2500 kcal this would be 1806 mg/d. After a run-in period of 1 week on the controlled diet (“baseline”), individuals were randomized to take 8 sodium chloride capsules (i.e., 3.0 g = 130 mmol sodium), 8 potassium chloride capsules (i.e., 2.8 g = 72 mmol potassium), or 8 placebo capsules (cellulose) daily, for 4 weeks each. Individuals were weighed twice a week and, if needed, their energy intake was adjusted to keep body weight constant.

### Participants, eligibility, and consent

Eligible participants were 40 to 80 years old, with a fasting office SBP of 130 to 159 mm Hg. Exclusion criteria were diabetes mellitus, kidney diseases including chronic kidney disease (CKD), and gastrointestinal and liver diseases. Participants were also ineligible for participation if they were current smokers; had a body mass index >40 kg/m^2^; used medication that affected the cardiovascular system; used nutritional supplements; were on an energy-restricted or a medically prescribed diet; were women with premenopausal status or were taking oral contraceptives or estrogen replacement therapy; had unstable weight or used alcohol over 21 (women), or 28 (men) consumptions per week. Participants were recruited from December 2011 to April 2012.

### Measurements

Participants underwent venous blood sampling after the end of each treatment period at fixed time points of the day throughout the study, and collected 24 hours of urine. Serum, EDTA-plasma, and urine samples were stored at –80°C, and electrolytes were measured using routine laboratory procedures (Modular P, Roche Diagnostics, Mannheim, Germany). C-terminal FGF23 was determined in EDTA-plasma by enzyme-linked immunosorbent assay (ELISA, Immutopics, San Clemente, CA). The interassay coefficient of variation of this assay in our laboratory was < 2.5% (33). PTH and 25[OH]-vitamin D_3_, which are involved in renal phosphate handling (34), were measured in EDTA-plasma using an electrochemiluminescence immunoassay, and isotope dilution–online solid phase extraction liquid chromatography–tandem mass spectrometry, respectively.

Estimated glomerular filtration rate (eGFR) was calculated using the creatinine-based Chronic Kidney Disease Epidemiology Collaboration equation. Fractional excretion of phosphate and calcium were calculated as follows: Fractional excretion (phosphate/calcium) = Urinary phosphate/calcium [mmol/L] × serum creatinine [µmol/L] / plasma phosphate/calcium (mmol/L) × urinary creatinine [mmol/L] × 100. The kidney tubular maximum reabsorption / GFR (TmP/GFR) was calculated as a measure of the phosphate reabsorption threshold using the following formula (35): First tubular reabsorption of phosphate (TRP) was calculated: 1 – (urinary phosphate [mmol/L] × serum creatinine [μmol/L] / plasma phosphate [mmol/L] × urinary creatinine [mmol/L]). If TRP was ≤ 0.86 we used the following formula: TmP/GFR = plasma phosphate (mmol/L) × TRP. If TRP > 0.86, we used the following formula: TmP/GFR = α × TRP, let α = 0.3 × TRP / (1 – (0.8 × TRP)).

### Ethics

The Medical Ethics Committee of Wageningen University approved the study. The trial was registered at ClinicalTrials.gov (NCT01575041). The study was conducted from March to August 2012 at the research center of The Division of Human Nutrition and Health, Wageningen University, The Netherlands. All subjects gave written and oral informed consent.

### Statistics

Normally distributed data are presented as mean ± SD, whereas skewed data are presented as geometric mean with 95% confidence interval (CI). For each outcome measure, we used a mixed-effects model with covariance structure compound symmetry to estimate the effect of active treatment compared with placebo. Fixed effects were “treatment” and “period”; random effect was participant number. Variables were natural log transformed when appropriate, as assessed with histograms and Q-Q plots, and subsequently back-transformed. To correlate the changes of variables during potassium and sodium supplementation Spearman’s rho (rank) correlation was used to determine the associations between various delta variables. Mean percentage change of potassium or sodium supplementation compared with placebo was calculated by: ((potassium/sodium [variable] – placebo [variable]) / placebo [variable]) × 100. Findings were considered statistically significant when *P *< 0.05. Analyses were performed in SAS 9.3 (SAS Institute, Cary, NC), and SPSS software, version 23.0, for Windows (IBM, Armonk, NY).

## Results

### Population characteristics

The 36 participants were 65.8 years old (range, 47-80) and predominantly male (67%) with a body mass index of 27.2 ± 4.7 kg/m^2^. Participants had mildly elevated blood pressure at screening (average SBP, 145 ± 11 mm Hg; diastolic blood pressure [DBP], 81 ± 8 mm Hg). Baseline characteristics are presented in Table 1.

**Table 1.** Baseline Characteristics after Run-in Period

Variable	Overall Population (n = 36)
**Demographics**	
Male, n (%)	24 (67)
Age, y	66 ± 9
**Clinical measurements**	
BMI, kg/m^2^	27.2 ± 4.7
Body weight, kg	85.1 ± 18.4
Office SBP, mm Hg	133 ± 14
Heart rate, beats/min	60 ± 7
**Fasting blood parameters**	
Sodium, mmol/L	143.3 ± 1.6
Potassium, mmol/L	4.33 ± 0.34
Total cholesterol to HDL ratio, mmol	3.9 ± 1.0
Urea, mmol/L	5.4 ± 1.1
Creatinine, µmol/L	81 ± 13
eGFR, mL/min per 1.73 m^2^	79.4 ± 12.4
**Urinary parameters**	
Sodium excretion, mmol/24 h	91 ± 27
Potassium excretion, mmol/24 h	49 ± 13
ACR, mg/mmol	0.44 (0.30-0.63)

Abbreviations: ACR, albumin-to-creatinine ratio; BMI, body mass index; eGFR, estimated glomerular filtration rate; FGF23, fibroblast growth factor 23; HDL, high-density lipoprotein; SBP, systolic blood pressure. Data are presented as mean ± SD, geometric mean (95% confidence interval), or number (percentage).

### Effects of potassium supplementation on bone and mineral parameters

**Figure 1.**
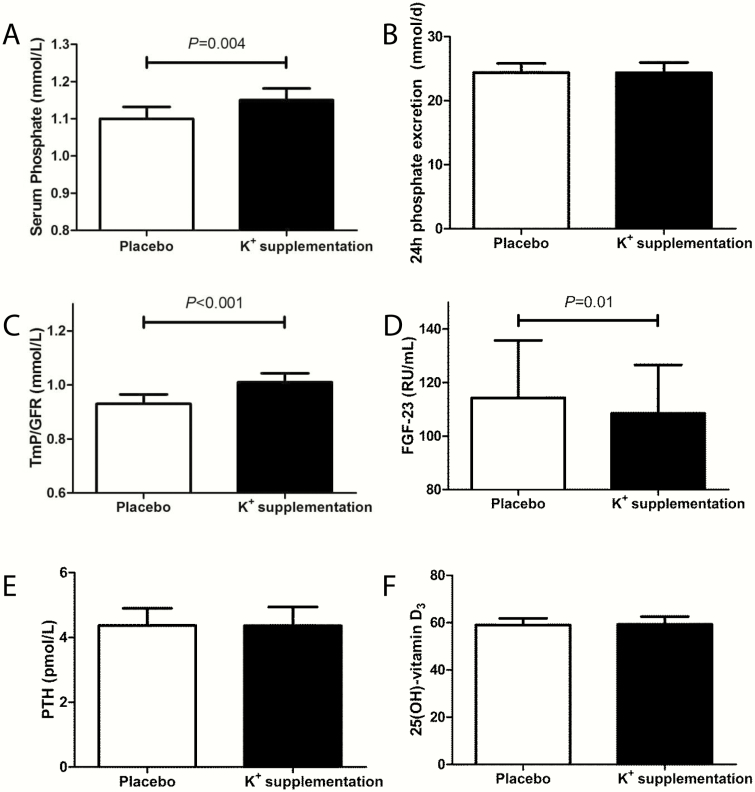
Effect of a 4-week period of potassium supplementation in (A) healthy prehypertensive adults on plasma phosphate (*P* = 0.004), (B) 24 hours urinary phosphate excretion (*P* = NS), and (C) TmP/GFR (*P *< 0.001). The rise of phosphate levels was paralleled by (D) a decrease in FGF23 (*P* = 0.01), (E) without effect on PTH (*P* = NS) or (F) 25[OH]-vitamin D_3_ (*P* = NS). Depicted are unadjusted means and standard error, or geometric means and 95% confidence intervals for FGF23 and PTH. Abbreviations: FGF23, fibroblast growth factor 23; NS, not significant; TmP/GFR, tubular maximum reabsorption of phosphate per glomerular filtration rate.

**Figure 2.**
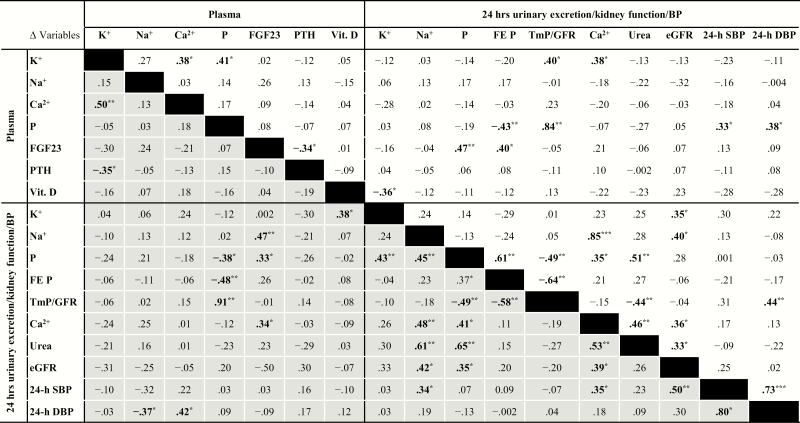
Spearman’s rho correlation coefficients for changes in blood and urine parameters in response to potassium (grey shaded area, lower left-hand side) or sodium (white area, upper right-hand side) supplementation vs placebo. ***P < 0.001, **P < 0.01, *P < 0.05. Abbreviations: Ca^2+^, calcium; eGFR, estimated glomerular filtration rate; FEP, fractional phosphate excretion; FGF23, fibroblast growth factor 23; K^+^, potassium; Na^+^, sodium; P, phosphate; TmP/GFR, tubular maximum reabsorption of phosphate per glomerular filtration rate; vit. D, 25(OH)-vitamin D_3_.

Potassium supplementation led to an increase in 24 hours urinary potassium excretion (from 55 ± 17 to 118 ± 32 mmol/24 hours), and also to a small increase in plasma potassium (from 4.29 ± 0.32 to 4.41 ± 0.30 mmol/L). FGF23 levels decreased during potassium supplementation compared with placebo (geometric mean: from 114.3 RU/mL [95% CI, 96.2-135.8, *P* = 0.01] to 108.5 RU/mL [95% CI, 93.0-126.6]) (Table 2 and Fig. 1D). The effect of potassium supplementation on FGF23 remained after adjustment for plasma phosphate (treatment effect: –0.06 [–0.11 to –0.02]), and showed similar trends in participants with plasma 25(OH)-vitamin D_3_ levels <50 nmol/L (N = 11, FGF23 from 103.4 [81.6-131.0] to 98.7 [78.2-124.6] RU/mL) vs participants with plasma 25(OH)-vitamin D_3_ levels >50 nmol/L (N = 24, FGF23 from 121.8 [97.6-152.1] to 114.9 [94.6-139.5] RU/mL). Compared with placebo, plasma phosphate concentration increased from 1.10 ± 0.19 to 1.15 ± 0.19 mmol/L (*P* = 0.004) (Table 2). The increase in plasma phosphate coincided with a decrease in fractional excretion of phosphate (from 15.8 ± 5.8 to 13.3 ± 4.2 %, *P *< 0.001) and an increase in the maximal phosphate tubular reabsorption, as reflected by the TmP/GFR (from 0.93 ± 0.21 to 1.01 ± 0.20, *P *< 0.001) (Table 2 and Fig. 1A, C). Furthermore, the change in TmP/GFR was correlated with the change in plasma phosphate (Fig. 2, *r*_*s*_ = 0.91, *P *< 0.001). The 24-hour urinary phosphate excretion did not change (Table 2 and Fig. 1B). Levels of 25(OH)-vitamin D_3_ and PTH also did not change after 4 weeks of potassium supplementation (Table 2 and Fig. 1E, F). Potassium supplementation did lead to a decrease in fractional calcium excretion (from 1.25 ± 0.50 to 1.11 ± 0.46 %, *P* = 0.03), and a nonsignificant lower trend in 24-hour urinary calcium excretion (from 4.28 ± 1.91 to 4.05 ± 2.15 mmol/24 hours, *P* = 0.3) (Table 2). The effect of potassium on fractional calcium excretion was relatively large, as reflected by a mean percentage change of –10.6% (Table 3). The change in FGF23 in response to potassium supplementation correlated with the change in urinary calcium excretion (*r*_*s*_ = 0.34, *P *< 0.05) (Fig. 2). Potassium supplementation did not, however, influence plasma calcium. As reported previously, 24-hour SBP and DBP decreased during potassium supplementation (24-hours SBP from 129 ± 14 to 126 ± 13 mm Hg, 24-hours DBP from 77 ± 8 to 75 ± 8 mm Hg) (31). Changes in FGF23 were not correlated with changes in blood pressure (Fig. 2). Potassium supplementation did not have an effect on eGFR compared with placebo (from 79.2 ± 11.6 mL/min per 1.73 m^2^ to 78.5 ± 11.7 mL/min per 1.73 m^2^).

**Table 2.** Mean Values of the Effects of 4 Weeks of Potassium or Sodium Supplementation on Bone and Mineral Parameters in a Randomized Placebo Controlled Trial in 36 Healthy Prehypertensive Adults

	Mean ± SD			Treatment Effect (95% CI)			
	Potassium	Placebo	Sodium	Potassium vs Placebo	*P*-value	Sodium vs Placebo	*P*-value
**Plasma**							
Potassium, mmol/L	4.41 ± 0.30	4.29 ± 0.32	4.18 ± 0.34	0.13 (0.05 to 0.20)	**0.002**	–0.10 (–0.18 to –0.02)	**0.01**
Sodium, mmol/L	142.7 ± 1.5	143.4 ± 1.2	143.8 ± 1.5	–0.7 (–1.1 to –0.2)	**0.004**	0.4 (–0.1 to 0.8)	0.10
Phosphate, mmol/L	1.15 ± 0.19	1.10 ± 0.19	1.06 ± 0.21	0.05 (0.02 to 0.09)	**0.004**	‒0.04 (‒0.08 to 0.00)	**0.03**
Calcium, mmol/L	2.34 ± 0.08	2.34 ± 0.06	2.33 ± 0.08	‒0.01 (‒0.03 to 0.02)	0.6	‒0.01 (‒0.04 to 0.01)	0.2
FGF23, RU/mL^*a*^	108.5 (93.5 - 125.9)	114.3 (96.8 - 135.0)	108.7 (92.3 - 128.1)	‒0.05 (‒0.09 to ‒0.01)	**0.01**	‒0.05 (‒0.09 to ‒0.01)	**0.02**
PTH, pmol/L^*a*^	4.36 (3.84 - 4.94)	4.37 (3.89 - 4.90)	4.37 (3.93 - 4.85)	0.00 (‒0.07 to 0.06)	0.9	0.00 (‒0.06 to 0.07)	0.9
**25(OH)-vitamin D**_**3**_ **, nmol/L** ^***b***^	59.0 ± 19.0	59.0 ± 16.9	58.3 ± 18.1	0.9 (‒1.6 to 3.3)	0.5	‒0.8 (‒3.3 to 1.7)	0.5
**Urine**							
Sodium excretion, mmol/24 h	96 ± 39	105 ± 40	203 ± 55	‒9 (‒25 to 8)	0.3	98 (81 to 114)	**<0.001**
Potassium excretion, mmol/24 h	118 ± 32	55 ± 17	53 ± 17	63 (55 to 71)	**<0.001**	‒2.2 (‒10 to 6)	0.6
Phosphate excretion, mmol/24 h	24.4 ± 9.6	24.4 ± 8.6	24.5 ± 7.3	‒0.02 (‒2.5 to 2.4)	0.99	0.05 (‒2.4 to 2.5)	0.98
Fractional excretion of phosphate, %	13.3 ± 4.2	15.8 ± 5.8	14.7 ± 4.5	‒2.5 (‒3.8 to ‒1.3)	**<0.001**	‒1.0 (‒2.3 to 0.2)	0.1
TmP/GFR, mmol/L	1.01 ± 0.20	0.93 ± 0.21	0.91 ± 0.22	0.07 (0.03 to 0.11)	**<0.001**	‒0.02 (‒0.06 to 0.01)	0.2
Calcium excretion, mmol/24 h	4.05 ± 2.15	4.28 ± 1.91	5.45 ± 2.51	‒0.24 (‒0.69 to 0.21)	0.3	1.16 (0.70 to 1.61)	**<0.001**
Fractional excretion of calcium, %	1.11 ± 0.46	1.25 ± 0.50	1.44 ± 0.54	–0.15 (–0.29 to –0.02)	**0.03**	0.19 (0.06 to 0.32)	**0.004**
Urea excretion, mmol/24 h	372 ± 103	355 ± 93	363 ± 120	16 (‒11 to 43)	0.2	7 (‒20 to 34)	0.6
**eGFR, mL/min per 1.73 m** ^**2**^	78.5 ± 11.7	82.7 ± 11.1	79.2 ± 11.6	‒0.4 (‒2.8 to 1.9)	0.7	3.5 (1.2 to 5.9)	**0.001**

Abbreviations: CI, confidence interval; eGFR, estimated glomerular filtration rate (Chronic Kidney Disease Epidemiology Collaboration [CKD-EPI]); FGF23, fibroblast growth factor 23; PTH, parathyroid hormone; SD, standard deviation; TmP/GFR, tubular maximum reabsorption of phosphate per glomerular filtration rate.

^*a*^Values are geometric mean and 95% CI for FGF23 and PTH, differences are changes in natural log-transformed variables.

^*b*^Because of 3 missing samples, analysis performed for 25(OH)-vitamin D3 was done as follows: potassium N = 36 (no missing data), placebo N = 35, sodium N = 34.

Bold *P*-values indicating significant result (*P* < 0.05).

**Table 3.** Mean Percentage Change of Potassium or Sodium Supplementation Compared with Placebo

Mean % Change Compared with Placebo	Potassium	Sodium
**Plasma**		
Potassium, mmol/L	**+3.1** ^***a***^	**−2.3** ^***a***^
Sodium, mmol/L	**−0.5** ^***a***^	+0.3
Phosphate, mmol/L	**+5.5** ^***a***^	**−3.4** ^***a***^
Calcium, mmol/L	−0.2	−0.5
FGF23, RU/mL	**−4.5** ^***a***^	**−4.2** ^***a***^
PTH, pmol/L	+1.3	+2.2
25(OH)-vitamin D_3_, nmol/L	+0.5	−2.0
**Urine**		
Sodium excretion, mmol/24 h	−20.7	**+43.2** ^***a***^
Potassium excretion, mmol/24 h	**+48.6** ^***a***^	−6.2
Phosphate excretion, mmol/24 h	+6.1	+8.3
Fractional excretion of phosphate, %	**−7.7** ^***a***^	+9.6
Calcium excretion, mmol/24 h	−1.9	**+33.9** ^***a***^
Fractional excretion of calcium, %	**−10.6** ^***a***^	**+21.6** ^***a***^
Urea excretion, mmol/24 h	+7.9	+2.9
**Other**		
TmP/GFR, mmol/L	**+10.8** ^***a***^	−1.3
eGFR, mL/min per 1.73 m^2^	−0.5	**+3.9** ^***a***^

Abbreviations: eGFR, estimated glomerular filtration rate; FGF23, fibroblast growth factor 23; PTH, parathyroid hormone; TmP/GFR, tubular maximum reabsorption of phosphate per glomerular filtration rate.

^*a*^Significant treatment effect of potassium or sodium supplementation compared with placebo.

### Effects of sodium supplementation on bone and mineral parameters

**Figure 3.**
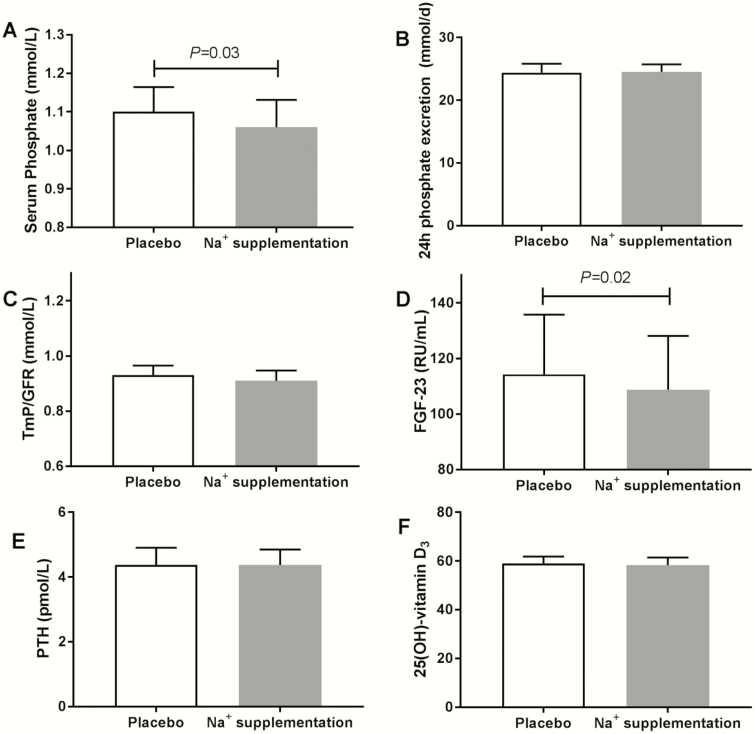
Effect of a 4-week period of sodium supplementation in healthy prehypertensive adults (A) on plasma phosphate (*P* = 0.03), 24 hours urinary phosphate excretion (B) (*P* = NS) (C) and TmP/GFR (*P * = NS). The rise of phosphate levels was paralleled by (D) a decrease in FGF23 (*P* = 0.02), (E) without effect on PTH (*P* = NS) or (F) 25[OH]-vitamin D_3_ (*P* = NS). Depicted are unadjusted means and standard error, or geometric means and 95% confidence intervals for FGF23 and PTH. Abbreviations: FGF23, fibroblast growth factor 23; TmP/GFR, tubular maximum reabsorption of phosphate per glomerular filtration rate.

Sodium supplementation increased urinary sodium excretion (from 105 ± 40 to 203 ± 55 mmol/24 hours, *P *< 0.001), without a change in plasma sodium. After 4 weeks of sodium supplementation, FGF23 levels decreased compared with placebo (108.7 RU/mL [95% CI, 92.3-128.1] vs 114.3 RU/mL [95% CI, 96.2-135.8, *P* = 0.02]) (Table 2 and Fig. 3D). Plasma phosphate was also significantly decreased compared with placebo supplementation (from 1.10 ± 0.19 to 1.06 ± 0.21 mmol/L) (Table 2 and Fig. 3A). Sodium supplementation did not significantly influence TmP/GFR, 24 hours urinary phosphate excretion, or fractional phosphate excretion (Table 2 and Fig. 3B, C). Yet, the change in FGF23 was positively correlated with the change in urinary (fractional) phosphate excretion (*r*_*s*_ = 0.47, *P *< 0.01; *r*_*s*_ = 0.40, *P *< 0.05) and it was inversely correlated with the change in PTH levels (*r*_*s*_ = –0.34, *P *< 0.05) (Fig. 2). Sodium supplementation led to an increased urinary calcium excretion (from 4.28 ± 1.91 to 5.45 ± 2.51 mmol/24 hours) and fractional calcium excretion (from 1.25 ± 0.50 to 1.44 ± 0.54%) compared with placebo (Table 2) with a mean change of +33.9% and +21.6%, respectively (Table 3). Other bone and mineral parameters were not significantly different between sodium supplementation and placebo (Table 2 and Fig. 3E, F). During sodium supplementation, 24-hour SBP increased from 129 ± 14 to 122 ± 15 mm Hg and 24-hour DBP increased from 77 ± 8 to 79 ± 9 mm Hg) (31); there were no correlations with changes in FGF23 (Fig. 2). In response to sodium supplementation, eGFR increased significantly from 79.2 ± 11.6 mL/min per 1.73 m^2^ to 82.7 ± 11.6 mL/min per 1.73 m^2^ (*P* = 0.003) compared with placebo, but this change did not correlate with a change in FGF23 (*r*_*s*_ = –0.07, *P* = NS) (Fig. 2).

## Discussion

In this post hoc analysis of a randomized, placebo-controlled crossover trial with dietary control, both potassium and sodium supplementation reduced FGF23 levels. During potassium supplementation, this reduction was accompanied by a concomitant increase of renal phosphate reabsorption and plasma phosphate levels, without an effect on PTH or 25(OH)-vitamin D_3_. In contrast, during sodium supplementation, the reduction of FGF23 was accompanied by a decrease of plasma phosphate. Furthermore, potassium supplementation decreased fractional calcium excretion and sodium supplementation led to an increase of urinary and fraction calcium excretion. Together, these findings suggest that sodium and potassium intake have differential effects on mineral metabolism, even though the underlying mechanisms seem complex and are not fully elucidated by the current study.

In the original study, 4 weeks of potassium supplementation decreased blood pressure which was mitigated by vasopressin, stimulation of renin and aldosterone, and an increased heart rate (31, 36). The current study shows that higher potassium intake, independent of phosphate or protein intake, decreased FGF23 levels. This is in line with findings from a previous study showing that individuals consuming a potassium-poor Western diet display higher FGF23 levels, and that potassium excretion was inversely associated with FGF23 (25). Effects of potassium on phosphate metabolism have been reported previously in preclinical and clinical studies. Potassium supplementation was shown to stimulate phosphate reabsorption in rats (37), presumably dependent on PTH. Moreover, 1 study in healthy adults found that potassium bicarbonate and potassium chloride changed the set point of phosphate reabsorption, resulting in higher plasma phosphate levels (38). Accordingly, in our study, potassium supplementation also increased TmP/GFR, and decreased the fractional excretion of phosphate, resulting in a higher plasma phosphate level, whereas PTH and 25(OH)-vitamin D_3_ remained unchanged. These findings suggest that potassium supplementation decreased plasma FGF23, resulting in increased phosphate reabsorption in the kidney and higher plasma phosphate.

The extracellular matrix in bone has a 5-fold higher potassium concentration compared with extracellular fluid, a gradient that is maintained by active transport mechanisms (39). We postulate that bone may serve as a buffer for an increase in dietary potassium intake, to which osteocytes may respond by reducing FGF23 production. Future studies should address the impact of dietary potassium supplementation on both FGF23 and plasma phosphate in osteocytes, animal models, and in specific patient groups such as CKD patients (40). CKD patients are at risk for mineral bone disorders, which is linked to the derangements of 1,25[OH]-vitamin D_3_, PTH, and FGF23 (41). FGF23 levels progressively increase with declining kidney function, and patients with end-stage kidney disease display the highest levels of FGF23 (42, 43). A large number of epidemiological studies have linked a higher FGF23 level with adverse cardiovascular outcomes independent of kidney function and established cardiovascular risk factors (21, 44). At the same time, higher potassium intake is associated with better outcomes in various populations, including CKD patients (7, 45, 46). In addition to reducing blood pressure (47), FGF23 reduction might be an additional pathway, through which potassium supplementation could lower the risk of adverse outcomes in CKD patients.

Potassium chloride or potassium bicarbonate have been shown to reduce urinary calcium excretion in some (48), but not all previous studies (27, 49–51). Furthermore, a recent elegant study in mice strengthened this observation by showing that mice receiving a low potassium and high sodium diet displayed an increased urinary calcium excretion compared to mice with a normal potassium and high sodium diet (52). The authors proposed that the effect of low potassium on urinary calcium excretion is mediated by the thick ascending limb of Henle’s loop on top of sodium-dependent calcium reabsorption in the proximal tubule. Furthermore, potassium acts as a thiazide diuretic by inhibiting the sodium-chloride cotransporter in the distal collecting duct, which in turn also lowers calcium excretion (53). Another study suggested that urinary calcium reabsorption is influenced directly by FGF23 (54), although the current study does not allow to draw a conclusion on a potential cause-effect relationship. Several studies have shown that supplementation of potassium alkali could decrease bone resorption markers and increase calcium balance (27, 49). In our controlled diet study, we were not able to assess if the decrease of fractional calcium excretion could lead to an improvement of bone health. However, increased urinary calcium excretion might reflect lower bone density and a higher risk of fractures (55). Also, in some studies, high FGF23 has been associated with poor bone health (56, 57), whereas high dietary potassium intake has been associated with improved bone health (27, 28).

To our knowledge, 2 previous studies addressed the effect of sodium interventions on FGF23. We previously found no effect of low sodium intake on FGF23 in patients with CKD, and also no effect of saline infusion in patients with hypertension (58). On the other hand, in a study in healthy adults, high salt intake decreased FGF23, whereas other bone and mineral parameters were not investigated in that study (59). In the current study, we found that sodium supplementation decreased FGF23 levels, which was not accompanied by a change in fractional phosphate excretion or TmP/GFR. In contrast with the effect of potassium, sodium supplementation led to a decrease in plasma phosphate, suggesting a different sequence of events. Sodium supplementation could lead to lower plasma phosphate through an increase in extracellular fluid volume (60), as supported by the observed increased in volume markers (36). The lower FGF23 levels during sodium supplementation may be secondary to lower plasma phosphate, reflecting an attempt to retain phosphate to maintain phosphate balance (14). A comprehensive study in mice showed that FGF23 directly regulates sodium homeostasis by increasing sodium-chloride cotransporter membrane abundance in the distal convoluted tubule, suggesting cross-talk between FGF23 and sodium homeostasis (61). As an alternative explanation, in the current study, sodium supplementation increased blood pressure and increased eGFR (31). This might also explain the decline in FGF23 (62), although we could not demonstrate a significant association between the change in FGF23 and change in 24-hour blood pressure or eGFR during either intervention (Fig. 2).

Our finding that sodium supplementation strongly induces hypercalciuria is well in line with several previous studies, also showing that high sodium intake contributes to the development and progression of osteoporosis and kidney stones (55, 63, 64). In clinical practice, low salt intake is recommended to lower the risk of recurring calcium-containing kidney stones (65).

Strengths of this study include the 90% controlled diet and the double-blinded placebo-controlled design of the original study, as well as the crossover design that increased statistical power. In this highly controlled diet setting, introduction of a single mineral could affect the bone and mineral parameters, suggesting that the effect is indeed induced by that mineral. Limitations of this study include the limited sample size and the relatively short follow-up. The study did not include a washout period between the interventions and, although limited data are available about lasting effects of potassium or sodium on bone and mineral parameters, carryover effect could not be excluded. Of note, baseline urinary potassium and sodium excretion is lower than urinary potassium and sodium excretion during the placebo period. Still, during the potassium or sodium supplementation period, a significant difference with the placebo period was observed in urinary potassium and sodium excretion, respectively, indicating that the effect of potassium and sodium supplementation is higher compared with placebo supplementation. This study was conducted on otherwise healthy prehypertensive adults and the observed results cannot be extrapolated to other patient populations. Finally, we did not have data on active vitamin D (1,25(OH)-vitamin D_3_), which might have elucidated some of the mechanisms driving our results.

In conclusion, we demonstrate in a post hoc analysis of a dietary controlled trial that potassium and sodium supplementation specifically influence calcium-phosphate metabolism, among others, by influencing FGF23. The interpretation of the interplay between sodium, potassium, and calcium-phosphate homeostasis remains highly complex. Our results provide a basis to further study the clinical impact of these interactions in specific patient populations in which potassium and mineral metabolism are deregulated, including patients with CKD.

## Abbreviations

25(OH)-vitamin D3: 25-hydroxy vitamin D3
CI: confidence interval
CKD: chronic kidney disease
DBP: diastolic blood pressure
eGFR: estimated glomerular filtration rate
FGF23: fibroblast growth factor 23
GFR: glomerular filtration rate
TmP: tubular maximum reabsorption
TRP: tubular reabsorption of phosphate
SBP: systolic blood pressure.

## Additional Information

***Disclosure Summary:*** No potential conflicts of interest relevant to this article were reported.

***Data Availability:*** The datasets generated during and/or analyzed during the current study are not publicly available but are available from the corresponding author on reasonable request.
